# One new species of the subgenus Hexatoma (Eriocera) Macquart (Diptera, Limoniidae) from China with a key to Chinese species

**DOI:** 10.3897/zookeys.477.7570

**Published:** 2015-01-26

**Authors:** Qiu-Lei Men, Dao-Ping Yu

**Affiliations:** 1 School of Life Sciences, Anqing Normal University, Anqing, Anhui 246011, P.R. China

**Keywords:** Nematocera, Crane flies, Tipuloidea, taxonomy

## Abstract

One new species of the subgenus *Eriocera* Macquart, 1838, Hexatoma (Eriocera) cleopatroides Men, **sp. n.** (Southern China: Anhui) is described and illustrated. A key to all of 78 known species from China in the subgenus is provided, which was solely based on literatures. The new species is similar to Hexatoma (Eriocera) cleopatra Alexander, 1933, but distinguishes from the latter by the prescutum entirely black with two ill-defined gray stripes, by the legs with fore and middle femora brown in basal half, black in apical half, with hind femora brown in basal one-fourth, and by the wings with cells c and sc more yellowish brown than the ground color.

## Introduction

*Eriocera* Macquart, 1838 was originally established as a genus with a single species *Limnobia nigra* Wiedemann. Currently, Eriocera is considered a subgenus of the genus *Hexatoma* Latreille, 1809, in which five other subgenera are also included, *Hexatoma* Latreille, 1809, *Cladolipes* Loew, 1865, *Coreozella* Enderlein, 1936, *Euhexatoma* Alexander, 1936 and *Parahexatoma* Alexander, 1951. It is characterized by the following characters: medium to large size; palpus with segments equilong; rostrum protruded obliquely from the vertex to the end; anterior vertex often with a greatly enlarged or variously modified tubercle; antenna filiform, with six to eight segments in male, eight to eleven in female, scape quite thick, slightly elongated, cylindrical, pedicel very short, first flagellomere slightly longer than the length of scape and pedicel together; legs with spur formula 2:2:2; wings uniformly tinged with black, gray or brown, sometimes with a conspicuous brown or yellow cross band before cord, rarely subhyaline or hyaline, Rs very long, M with at least three branches, cell m_1_ present in Palaearctic, Nearctic and oriental species, and absent in Neotropical species; male hypopygium with two gonostyli, the outer gonostylus with a sharp hook apically, inner gonostylus fluted; ovipositor elongated (Alexander 1948, Savchenko 1986). The subgenus *Eriocera* comprises 565 species (Oosterbroek 2014) and is very well represented in Oriental, Palaearctic, Nearctic and Neotropical regions, and rarely occurs in Australian and Afrotropical regions. The Chinese fauna of subgenus *Eriocera* is extremely rich with 77 members recorded (Alexander 1920, 1923a, 1923b, 1925, 1927, 1930, 1931, 1932, 1933a, 1933b, 1934, 1936, 1937a, 1937b, 1938a, 1938b, 1938c, 1940, 1941, 1942, 1943, 1945, 1949a, 1949b, 1949c, Edwards 1916, 1921, Enderlein 1912, Osten-Sacken 1881, Riedel 1913, Yang 1999, Walker 1848, Westwood 1836, Wiedemann 1828). The Chinese species of the subgenus are mainly distributed in southern China, rarely reported from northern China (Yang 1999). There is little published research on immature stages of subgenus *Eriocera*, for which only five species were reported (Alexander 1915a, Alexander and Lloyd 1914).

While sorting and identifying crane flies collected from Yaoluoping National Nature Reserve, Anhui Province, China, we found one new species of the subgenus *Eriocera*. In the present paper, we describe and illustrate the new species. In addition, a key for separating the known species from China is provided.

## Material and methods

The specimens examined in this study were collected during scientific exploration in Yaoluoping National Nature Reserve, Anhui Province, undertaken by undergraduates and author. The genital segments of the specimens were removed and soaked in 10% NaOH overnight and observed or drawn in glycerin jelly using a Leica MZ125 (Leica, Germany) stereomicroscope. The measurements were made with the aid of a digital caliper. All measurements are in millimeters (mm). The terminology and methods of description and illustration follow that of Alexander and Byers (1981), and Ribeiro (2006).

In the present study, no specimens of the other known species were available for examination. However, there is no doubt concerning the identity of those species because the descriptions and illustrations were very clear and detailed. The examined specimens of the new species are deposited in the animal specimen room, School of Life Sciences, Anqing Normal University, Anqing, Anhui Province, China. The key was constructed from the literatures.

## Taxonomy

### Subgenus Eriocera

Taxon classificationAnimaliaDipteraLimoniidae

Macquart, 1838

Eriocera Macquart 1838: 74; Brunetti 1912: 530; Edwards 1921: 67; Alexander 1933a: 148; Alexander 1948: 529; Alexander 1966: 415.
Caloptera Guerin-Méneville 1831: 20.
Evanioptera Guerin-Méneville 1838: 287.
Allartmia Loew 1850: 36.
Oligomera Doleschall 1857: 387.
Arrhenica Osten-Sacken 1860: 243.
Physecrania Bigot 1859: 123.
Penthoptera Schiner 1863: 220.
Androclosma Enderlein 1912: 34.
Globericera Matsumura 1916: 171.
Coreozelia Enderlein 1936: 22.

#### Key to species of the subgenus *Eriocera* from China

**Table:** 

1	Wings unicolor, without marks	**6**
–	Wings not unicolor, cells sc and c darker than ground color, or with marks	**3**
2	Prescutum with three stripes	**8**
–	Prescutum with four stripes (see Alexander 1933a: 155)	***omeiana* Alexander** (China: Sichuan)
3	Abdomen, not including hypopygium, unicolor	**15**
–	Abdomen, not including hypopygium, bicolor	**16**
4	Prescutum with stripes	**2**
–	Prescutum without stripes	**13**
5	Prescutum black	**7**
–	Prescutum gray (see Alexander 1949c: 448)	***fracida* Alexaner** (China: Fujian)
6	Abdomen, not including hypopygium, unicolor	**4**
–	Abdomen, not including hypopygium, bicolor	**5**
7	Head black	**9**
–	Head not black	**10**
8	Antenna brown throughout	**12**
–	Antenna with scape, pedicel and first flagellomeres black, the remainder missing (see Alexander 1933a: 158)	***nudivena* Alexander** (China: Sichuan-Xizang border)
9	The extreme cephalic and caudal portions of the prescutum with a capillary reddish brown median vitta (see Alexander 1923a: 298)	***abdominalis* (Alexander)** (China: Jiangxi)
–	Prescutum without such vitta	**11**
10	Head brownish gray; prescutum without stripe (see Osten-Sacken 1881: 406)	***moresa* (Osten-Sacken)** (China: Taiwan; Indonesia, Malaysia)
–	Head dull red; prescutum with four stripes (see Edwards 1916: 253)	***rubriceps* (Edwards)** (China: Taiwan)
11	Abdomen with segments two to five entirely orange (see Edwards 1921: 84)	***shirakii* (Edwards)** (China: Taiwan)
–	Abdominal tergites two to five apically with orange bands, the caudal margins remaining narrowly black (see Alexander 1938b: 350)	***scalator* Alexander** (China: Guangdong)
12	Cell m_1_ present; wings dark brown; head dark brown (see Alexander 1949c: 444)	***suberecta* Alexander** (China: Fujian)
–	Cell m_1_ absent; wings pale grayish; head gray (see Alexander 1933a: 159, Pl. 1, fig. 20)	***subpusilla* Alexander** (China: Sichuan)
13	Head yellow	**14**
–	Head black (see Riedel 1913: 273)	***nigrina* (Riedel)** (China: Taiwan)
14	Antenna distinctly shorter than body (see Alexander 1942: 183); basal deflection of CuA_1_ nearly its own length beyond base of discal cell (see Alexander 1942: 178, fig. 5)	***licens* Alexander** (China: Yunnan)
–	Antenna more than three times longer than body (see Wiedemann 1828: 56; Wulp 1895: 39, Pl. 2, fig. 6); basal deflection of CuA_1_ slightly beyond base of discal cell (see Alexander 1915b: Pl. 44, fig. 25)	***verticalis* (Wiedemann)** (China: Taiwan; Japan, Indonesia, Malaysia, Philippines)
15	Prescutum with stripes	**17**
–	Prescutum without stripes	**18**
16	Abdomen with tergites two to four yellow or orange	**31**
–	Abdomen with coloration not as above	**32**
17	Rostrum short, greatly reduced (see Alexander 1934: 330)	***diploneura* Alexander** (China: Sichuan)
–	Rostrum long, not reduced	**19**
18	Abdomen, not including hypopygium, black	**29**
–	Abdomen, not including hypopygium, dark brown or plumbeous	**30**
19	Prescutum with four stripes (see Alexander 1938a: 125)	***cantonensis* Alexander** (China: Jiangxi, Zhejiang, Guangdong)
–	Prescutum with less than four stripes	**20**
20	Prescutum with one middle stripe (see Alexander 1933a: 149)	***lanigrea* Alexander** (China: Sichuan-Xizang border)
–	Prescutum with three stripes	**21**
21	Abdomen, not including hypopygium, uniformly black	**23**
–	Abdomen, not including hypopygium, not black	**22**
22	Abdominal segments brownish gray; ovipositor with short hypovalva (see Alexander 1933a: 157)	***luteicostalis* Alexander** (China: Sichuan)
–	Abdominal segments brown; ovipositor with long hypovalva (see Alexander 1949a: 538)	***absona* Alexander** (China: Guangdong)
23	Head gray to brownish gray	**24**
–	Head black	**25**
24	Cell m_1_ lacking	**26**
–	Cell m_1_ present	**27**
25	Cell m_1_ a little shorter than its petiole; prescutum dark brown (see Alexander 1923a: 297)	***morula* (Alexander)** (China: Sichuan)
–	Cell m_1_ nearly twice its petiole; prescutum dull black (see Alexander 1927: 6, fig. 3)	***arrogans* (Alexander)** (China: Sichuan)
26	Mesonotum and pleura gray	**28**
–	Mesonotum dark brown; pleura black (see Alexander 1943: 179)	***gressittiana* Alexander** (China: Guangdong)
27	Wings with strong rufous tinge; m-cu not far beyond the fork of M; mesonotum blackish (see Alexander 1927: 4)	***fumidipennis* Alexander** (China: Sichuan)
–	Wings with strong black tinge; m-cu a little more than one-half its length beyond the fork of M; mesonotum gray (see Alexander 1925: 87, 88, fig. 1)	***rufipennis* Alexander** (China: Guangdong)
28	Prescutum with stripes black; wings tinged with brown (see Alexander 1938b: 347)	***toi* Alexander** (China: Hainan)
–	Prescutum with stripes dark gray; wings tinged with yellow (see Alexander 1949b: 202)	***canescens* Alexander** (China: Guangdong)
29	Cell r_4_ with a longitudinal or oblique vein, connecting R_5_ with M_1+2_; head black (see Alexander 1937a: 82, fig. 12)	***pieli* Alexander** (China: Zhejiang)
–	Cell r_4_ without such vein; head orange (see Alexander 1940: 26, 27, fig. 14)	***pterotricha* Alexander** (China: Jiangxi)
30	Cell m_1_ absent, m-cu at near two-thirds the length of cell first m_2_ (see Alexander 1945: 28, fig. 5)	***elevata* Alexander** (China: Guangdong)
–	Cell m_1_ present, m-cu at or just before midlength of cell first m_2_ (see Alexander 1937b: 388, Pl. 1, fig. 15)	***quadriatrata* Alexander** (China: Jiangxi)
31	Prescutum with stripes	**33**
–	Prescutum without stripe	**34**
32	Abdominal tergites two to seven brilliant purplish blue, the caudal margins of segments dull black (see Alexander 1936: 131)	***tuberculata* Alexander** (China: Hainan)
–	Abdominal tergites with coloration not as above	**38**
33	Prescutum with four stripes (see Alexander 1931: 359)	***caesarea* (Alexander)** (China: Sichuan)
–	Prescutum with less than four stripes	**36**
34	Cell m_1_ present (see Alexander 1927: 3, fig. 2)	***grahami* (Alexander)** (China: Sichuan)
–	Cell m_1_ absent	**35**
35	Ovipositor with genital shield orange; wings with a large, pale area in cells cu at near middle (see Alexander 1941: 414)	***regina kiuhuana* Alexander** (China: Anhui)
–	Ovipositor with genital shield black; wings without pale area in cells cu (see Alexander 1930: 73)	***platysoma* (Alexander)** (China: Sichuan)
36	The extreme cephalic portion of prescutum variegated by reddish on either side of median vitta; legs with femora entirely dark brown (see Alexander 1933a: 164)	***cleopatra* Alexander** (China: Sichuan)
–	Prescutum without such reddish portions; legs with femora yellow or brownish yellow basally, blackened apically	**37**
37	Hypopygium reddish yellow; prescutum with three stripes (see Alexander 1933a: 163)	***pyrrhopyga* Alexander** (China: Anhui, Fujian)
–	Hypopygium black; prescutum with two stripes	***cleopatroides* Men, sp. n.**
38	Prescutum with stripes	**39**
–	Prescutum without stripes	**40**
39	Prescutum with only one middle stripe	**41**
–	Prescutum with more than one stripe	**42**
40	Head black	**58**
–	Head not black	**59**
41	Wings strongly blackened; head deep reddish; abdominal sternites orange (see Alexander 1949c: 446, 447)	***eos* Alexander** (China: Fujian)
–	Wings with strong fulvous-brown tinge; head brown; abdominal sternites obscure yellow (see Alexander 1933a: 151)	***mediofila* Alexander** (China: Sichuan-Xizang border)
42	Prescutum with three stripes	**43**
–	Prescutum with four stripes	**44**
43	Abdominal segments reddish brown with caudal borders narrowly gray (see Alexander 1949c: 449)	***carinivertex* Alexander** (China: Fujian)
–	Abdominal segments with coloration not as above	**45**
44	Head black	**54**
–	Head brown or brownish gray	**55**
45	Cell m_1_ present	**46**
–	Cell m_1_ absent	**47**
46	Antenna entirely dark brown; wings brown, wing-apex broadly darker brown (see Alexander 1923b: 255)	***muiri* (Alexander)** (China: Guangdong)
–	Antenna entirely black; wing-apex without darker tinge	**48**
47	Legs brownish black or black throughout	**49**
–	Legs with each segment in different colors or bicolor in same segment	**50**
48	Halteres entirely dark brown; head brownish gray; abdominal sternites yellow (see Alexander 1933a: 150, 151)	***tibetana* Alexander** (China: Sichuan-Xizang border)
–	Halteres with stem brownish black, knob black; head blackish; abdominal sternites fulvous (see Alexander 1933b: 150, 151)	***hemicera* (Alexander)** (China: Sichuan)
49	Wings with two yellow blotches in cells r (see Alexander 1923b: 256)	***terryi* (Alexander)** (China: Guangdong)
–	Wings without yellow blotches in cells r	**51**
50	Head black	**52**
–	Head yellow	**53**
51	Antenna 10-segmented in female; Rs about one-half longer than R (see Alexander 1938b: 351, fig. 11)	***tinkhami* Alexander** (China: Guangdong)
–	Antenna 11-segemnted in female; Rs about one-third longer than R (see Alexander 1938b: 352, fig. 12)	***hoffmanni* Alexander** (China: Guangdong)
52	Abdominal tergites with basal rings plumbeous (see Alexander 1938a: 124)	***ambrosia* Alexander** (China: Guangdong)
–	Abdominal tergites with basal rings iridescent (see Alexander 1949a: 536)	***ambrosia angustinigra* Alexander** (China: Fujian)
53	Wings strongly tinged with yellow brown, cells c and sc light brown (see Alexander 1934: 330)	***minensis* Alexander** (China: Sichuan)
–	Wings nearly hyaline, cells c and sc yellowish (see Yang 1999: 41)	***flavimarginata* (Yang)** (China: Henan)
54	Antenna with each segment in different colors	**56**
–	Antenna entirely dark brown (see Alexander 1920: 259)	***lygropis* (Alexander)** (China: Taiwan)
55	Abdomen reddish-brown; cell m_1_ not present (see Alexander 1938b: 354, fig. 13)	***monoleuca* Alexander** (China: Hainan)
–	Abdomen yellow; cell m_1_ present (see Alexander 1940: 24, fig. 13)	***pieliana* Alexander** (China: Zhejiang)
56	Cell m_1_ present (see Alexander 1938b: 354, fig. 14)	***bifenestrata* Alexander** (China: Hainan)
–	Cell m_1_ not present (see Alexander 1932: 123, fig. 15; Alexander 1937a: 85, fig. 14)	**57**
57	Antenna with scape and pedicel dark brown, flagellum yellowish brown, the outer segments again darkened; legs with femora brownish black (see Alexander 1932: 123)	***kelloggi* (Alexander)** (China: Fujian, Guangdong)
–	Antenna with scape and pedicel black, the latter more reddish at apex, basal three flagellomeres yellow, the remainder passing into black; legs with femora yellow, the apex narrowly and abruptly blackened (see Alexander 1937a: 85)	***posticata* Alexander** (China: Zhejiang)
58	The lateral margins of abdomen with marks	**60**
–	The lateral margins of abdomen without marks	**61**
59	Wings with a cross band before cord	**74**
–	Wings without cross band (see Alexander 1938a: 120)	***insidiosa* Alexander** (China: Guangdong)
60	Wings with a cross band before cord (see Alexander 1938c: 3)	***kiangsuana* Alexander** (China: Jiangxi)
–	Wings without cross band	**62**
61	Legs with femora entirely black	**63**
–	Legs with femora not entirely black	**64**
62	Head deep orange; legs with different colors in different segments; wings with a strong brownish yellow suffusion (see Alexander 1937a: 79)	***sycophanta* Alexander** (China: Jiangxi)
–	Head bright yellow; legs black throughout; wings with a strong blackish suffusion (see Alexander 1937a: 80)	***kolthoffi* Alexander** (China: Jiangsu)
63	Abdomen black, tergites two, four and five with leaden basal bands (see Edwards 1921: 87)	***sinensis* (Edwards)** (China: Sichuan)
–	Abdomen with coloration not as above	**65**
64	Antenna entirely black	**70**
–	Antenna not uniformly colored	**71**
65	Antenna entirely dark brown	**66**
–	Antenna entirely black	**67**
66	Wings gray (see Alexander 1949c: 445)	***celestissima* Alexander** (China: Guangdong)
–	Wings dark brown	**68**
67	Abdominal tergite three uniformly black (see Alexander 1927: 4)	***cybele* (Alexander)** (China: Sichuan)
–	Abdominal tergite three bicolor	**69**
68	Sc_1_ equal to the deflection of CuA_1_, basal deflection of R_4+5_ about 2.5 times longer than r-m (see Alexander 1923a: 295)	***davidi* (Alexander)** (China: Sichuan, Fujian, Jiangxi, Zhejiang, Guangdong)
–	Sc_1_ shorter than the deflection of CuA_1_, basal deflection of R_4+5_ more than three times longer than r-m (see Alexander 1923a: 296)	***hilpoides* (Alexander)** (China: Sichuan)
69	Hypopygium orange; cell m_1_ present (see Edwards 1921: 88)	***chrysomela* (Edwards)** (China: Fujian, Jiangxi, Guangdong)
–	Hypopygium black; cell m_1_ not present (see Alexander 1937a: 83, fig. 13)	***regina* Alexander** (China: Anhui, Jiangxi)
70	Abdominal segments with silvery luster on anterior borders (see Walker 1848: 79)	***hilpa* (Walker)** (China: Anhui, Zhejiang, Guangdong)
–	Abdominal segments without silvery luster	**72**
71	Legs with coxae and trochanters black, remainder of legs dark brown; cell m_1_ present; hypopygium orange (see Alexander 1942: 183, fig. 4)	***sincera* Alexander** (China: Yunnan)
–	Legs brownish black throughout; cell m_1_ absent; hypopygium black (see Alexander 1923b: 256)	***submorosa* (Alexander)** (China: Guangdong)
72	Wings with an oblique whitish hyaline cross band before cord	**73**
–	Wings without cross band (see Enderlein 1912: 42)	***sauteriana* (Enderlein)** (China: Taiwan)
73	Legs with femora entirely yellow (see Alexander 1938a: 122)	***celestia* Alexander** (China: Guangdong)
–	Legs with femora yellow basally, the tip gradually and much more broadly blackened (see Alexander 1949a: 536)	***celestia maligna* Alexander** (China: Guangdong)
74	Antenna uniformly colored	**75**
–	Antenna with different colors in different segments	**76**
75	Head blackish gray; antenna entirely black	**77**
–	Head plumbeous; antenna entirely brown (see Alexander 1949c: 450)	***urania* Alexander** (China: Guangdong)
76	Abdomen reddish brown; prescutum reddish brown (see Alexander 1923b: 254)	***praelata* (Alexander)** (China: Guangdong)
–	Abdomen black; prescutum black (see Alexander 1936: 131)	***hirtithorax* Alexander** (China: Hainan)
77	Wings bright orange-yellow at the base (see Westwood 1836: 681)	***nepalensis* (Westwood)** (China: Sichuan, Guangdong; India, Malaysia, Nepal)
–	Wings with the base not brightened (see Alexander 1923b: 255)	***obliqua* (Alexander)** (China: Jiangxi, Guangdong)

### Hexatoma (Eriocera) cleopatroides

Taxon classificationAnimaliaDipteraLimoniidae

Men sp. n.

http://zoobank.org/773B8999-ACE1-4AD4-A223-C1855120DBE1

Figs 1–3 , 4–6

#### Diagnosis.

Antennal flagellum yellow. Head and thorax black, prescutum with two ill-defined grayish stripes. Wings tinged with light brown, cells c and sc more yellowish brown than ground color, wing-apex blackish, the basal half except extreme base also blackish. Abdominal segments two to four orange.

#### Description.

Body length: male 15.5–16.5 mm (n=2), female 18.3 mm (n=1). Wing: male 15.5–17.5 mm (n=2), female 15.2 mm (n=1). Antenna: male 4.5 mm, female 4.2 mm.

**Figures 1–3.**
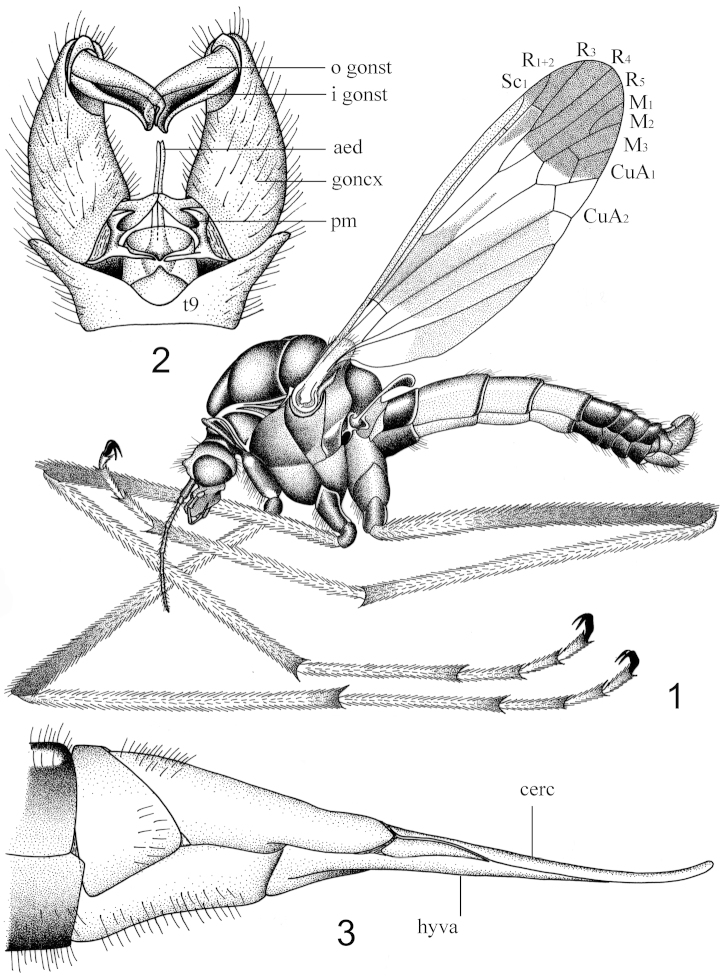
Hexatoma (Eriocera) cleopatroides Men, sp. n. **1** habitus of male adult, lateral view **2** hypopygium, dorsal view **3** ovipositor, lateral view. Abbreviations: aed=aedeagus, cerc=cercus, goncx=gonocoxite, hyva=hypovalva, i gonst=inner gonostylus, o gonst=outer gonostylus, pm=paramere, t=tergite.

Head. Rostrum dark brown with dark brown nasus. Vertex and occiput blackish. Setae on head black. Antenna 7-segmented in both sexes, relatively short, if bent backward not extending to the root of halteres (Fig. 1); scape black, elongated; pedicel black, very short; flagellum yellow, the first flagellomere longest, the remainder progressively shortened (Fig. 1). Verticils black, shorter than flagellomeres. Palpi black, the setae on palpi black. Tubercle enlarged (Fig. 1).

Thorax. Pronotum black. Prescutum black with two ill-defined grayish stripes. Scutum and scutellum black. Pleura deep brown. Setae on thorax mainly distributed on the lateral side of the prescutum. Coxae black; trochanters black; fore and middle femora brown in basal half, black in apical half (Fig. 1); hind femora brown in basal one-fourth, the remainder black (Fig. 1); tibiae dark brown, black at apex; tarsi black. Tibia spurs black with 2-2-2 in number. Setae on coxae and trochanters long, black, the remainder relatively short. Wings with ground color light brown, more yellowish brown in cells c and sc; stigma inconspicuous; wing with apex blackish, the basal half of wing except the extreme base also blackish (Fig. 1). Sc ending beyond the fork of R_2+3+4_; R_2+3_ distinctly shorter than R_3_; cell m_1_ present, asymmetrical, slightly longer than its petiole (Fig. 1). Halteres entirely black.

**Figures 4–6.**
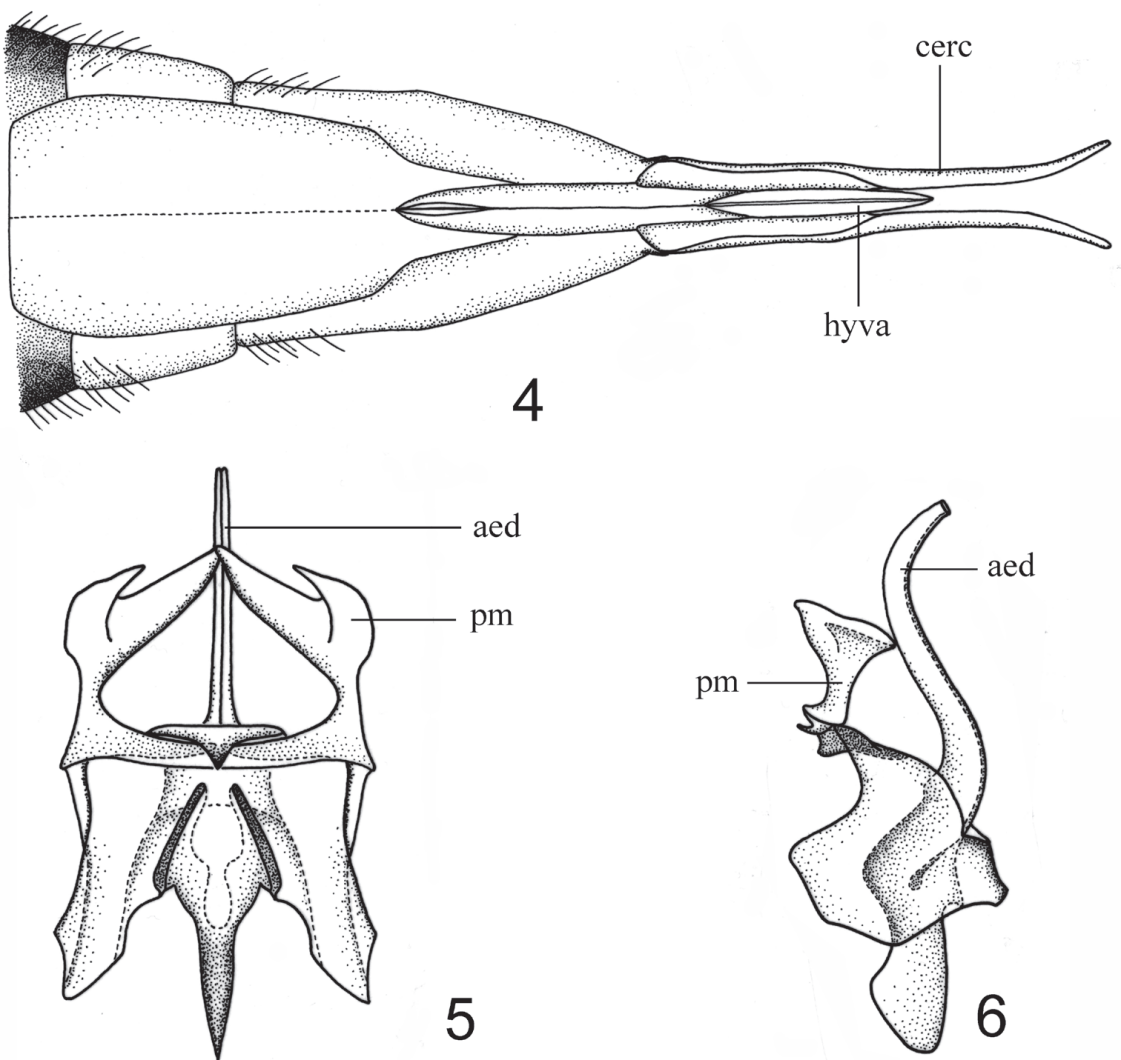
Hexatoma (Eriocera) cleopatroides Men, sp. n. **4** ovipositor, ventral view **5** aedeagal complex, dorsal view **6** aedeagal complex, lateral view. Abbreviations: aed=aedeagus, cerc=cercus, hyva=hypovalva, pm=paramere.

Abdomen. The first tergite black, narrowly ringed with orange at the caudal margin, the first sternite black also with orange stripe apically; tergites two to four orange, narrowly ringed with black apically, sternites uniformly orange; the remainder including hypopygium black in male (Fig. 1); the eighth to tenth tergites orange in female, ovipositor with cercus long and straight, basally brown and gradually passing into orange apically, hypovalva relatively long, orange (Figs 3, 4). Hypopygium with outer gonostylus slender, dark brown, the terminal spine decurved (Fig. 2); inner gonostylus dark brown, thick, fluted (Fig. 2); paramere bifid, curved inwardly, forming two triangular lobes, the ventral one larger than the dorsal one (Figs 2, 5, 6); aedeagus tubular, S-shaped in lateral view (Fig. 6).

#### Type material.

**Holotype** male. Pinned specimen. China: Anhui Province, Yuexi County, Yaoluoping National Nature Reserve, 31°2.123'N, 116°6.290'E, 1000m, 16 Aug. 2013, Z. K. Liu. Paratype. Pinned specimen. China: 1 male 1 female, Anhui Province, Yuexi County, Yaoluoping National Nature Reserve, 31°2.122'N, 116°6.209'E, 1000m, 17 Aug. 2013, Q. L. Men.

#### Distribution.

China (Anhui).

#### Remarks.

This new species is similar to another Chinese species Hexatoma (Eriocera) cleopatra from Sichuan by the color pattern of abdomen and wings. It can be easily distinguished from the latter by the prescutum entirely black with two ill-defined gray stripes (prescutum not entirely black, the extreme cephalic portion of prescutum variegated by reddish, with only one black median vitta in Hexatoma (Eriocera) cleopatra as described in Alexander 1933a); legs with fore and middle femora brown in basal half, black in apical half, with hind femora brown in basal one-fourth, the remainder black as shown in Fig. 1 (entirely dark brown in Hexatoma (Eriocera) cleopatra as described in Alexander 1933a); wings with cells c and sc more yellowish brown than the ground color as illustrated in Fig. 1 (cells c and sc not darker than the ground color in Hexatoma (Eriocera) cleopatra as described in Alexander 1933a).

#### Etymology.

The specific epithet is an adjective based on a name of a morphologically similar species, Hexatoma (Eriocera) cleopatra.

## Supplementary Material

XML Treatment for Eriocera

XML Treatment for Hexatoma (Eriocera) cleopatroides
